# Comments on the paper "A statistical assessment of differences and equivalences between genetically modified and reference plant varieties" by van der Voet et al. 2011

**DOI:** 10.1186/1472-6750-12-13

**Published:** 2012-04-25

**Authors:** Keith J Ward, Margaret A Nemeth, Cavell Brownie, Bonnie Hong, Rod A Herman, Regina Oberdoerfer

**Affiliations:** 1 Syngenta Ltd, Jealott's Hill, Bracknell, Berks, RG42 6EY, UK; 2 Monsanto Company, 800 N. Lindbergh Blvd., St Louis, MO 63167, USA; 3 P.O. Box 37160, Raleigh, NC 27627, USA (representing BASF; 4 Pioneer Hi-Bred International, Inc., 2450 SE Oak Tree Court, Ankeny, IA 50021, USA; 5 Dow AgroSciences LLC, 9330 Zionsville Road' Indianapolis, IN 46268, USA; 6 Bayer CropScience AG, Industriepark Höchst K801, 65926 Frankfurt, Germany

## Abstract

van der Voet et al. (2011) describe statistical methodology that the European Food Safety Authority expects an applicant to adopt when making a GM crop regulatory submission. Key to their proposed methodology is the inclusion of reference varieties in the experimental design to provide a measure of natural variation amongst commercially grown crops. While taking proper account of natural variation amongst commercial varieties in the safety assessment of GM plants makes good sense, the methodology described by the authors is shown here to be fundamentally flawed and consequently cannot be considered fit for purpose in its current form.

## Background

As the authors mention, van der Voet et al (2011) [1] is based on the European Food Safety Authority (EFSA)'s "Scientific Opinion on Statistical considerations for the safety evaluation of GMOs" [2] published on 1^st ^February 2010. The latter is the most recent version of a series of documents going back to June 2008 that detail the statistical methodology that EFSA expects an applicant to adopt when making a GM crop regulatory submission. During this period, EFSA has addressed some of the concerns that applicants have conveyed to them about the proposed methodology, but several of the most fundamental concerns have not been addressed and are present in the paper under discussion.

## Key Concerns

### Failure to take proper account of interactions in the statistical model

In a typical study for which the methodology is being proposed, replicated plots of GM plants, a conventional comparator and a number of reference varieties are laid out as a randomised complete block design at each of a number of different sites. Given this design, and adopting the term "Genotype" to represent the complete list of test entries (i.e. GMO, conventional comparator and reference varieties), the basic linear mixed model structure is as follows:

(1) $$Y_{ijk}=Mean+Site_{i}+Block_{ij}+Genotype_{k}+SitexGenotype_{ik}+e_{ijk}.$$

where i, j and k are the indices for site, block within site, and genotype respectively, *Y_ijk _*is the observed response at site i, block j for genotype k, *SitexGenotype_ik _*is the interaction between site and genotype, and *e_ijk _*is residual plot error. To embrace the authors' proposals it is necessary to partition some of the above terms into separate components but the basic structure still applies. For example, and as the authors explain, Genotype is separated into GenotypeGroup (a three-level fixed factor distinguishing GMO, comparator and the group of reference varieties as a whole) and Genotype-within-GenotypeGroup (a random factor representing genetic variation in the reference population). Partitioning the interaction between site and genotype into the corresponding components is the logical next step, supported by the argument that interaction with site may be different among the test entries than among reference varieties.

Crucially, the authors choose not to include any interaction terms in their model, and in our view omitting such interactions from the model has led to fundamentally flawed procedures.

To illustrate the importance of accounting for interactions, we first focus on the test comparing GMO and comparator means. By neglecting to include any terms for interaction in their model, the difference test proposed by the authors will always be based on an error term that includes all potential sources of interaction, as well as plot effects, with correspondingly large degrees of freedom. The consequence of this is not the same in all cases and instead depends on the relative sizes of the various sub-components of the site × genotype interaction. For example, in the not unrealistic situation in which both the site × test entry interaction and the site × reference variety interaction are non-zero and of broadly similar magnitude, the error term will be underestimated and the associated degrees of freedom will be far too high. As a consequence, the false positive error rate will tend to be higher than the nominal rate indicated and the confidence interval for the difference between means will be too narrow. Certain other arrangements will result in the proposed difference test being unduly conservative. In fact, the only situation for which the proposed difference test will behave as intended is when there are, in reality, no interactions whatsoever with site.

To illustrate the effect of ignoring interactions on the behaviour of the proposed difference test we conducted a small simulation exercise, details of which are given in the Methods section. For simplicity, data were generated under the basic model structure in equation (1) with a single component to represent the entire site × genotype interaction. This implicitly assumes that the magnitude of the site × test entry interaction is the same as the site × reference variety interaction, and while this may be a somewhat special case, the example is sufficient to demonstrate that, in general, the proposed methodology does not perform as intended.

Within the general framework of the difference test, taking proper account of the site × test entry interaction is not difficult conceptually, nor would it be difficult to implement in practice if the reference varieties were simply omitted from the analysis. Within the authors' specified framework, however, we have yet to find a way of adapting their SAS code that will result in the right degrees of freedom in all cases for even the most straightforward of EFSA-inspired experimental designs in which all test entries and reference varieties are included at all locations. (A design, incidentally, that would not be practicable on cost grounds). Conversely, how to take proper account of interactions with site in the authors' proposed tests of equivalence is conceptually far less obvious but clearly no less important. For example, should equivalence intervals reflect the fact that reference varieties are likely to perform differently at different locations? The way interactions are handled will have a direct bearing on the various standard errors and intervals that are central to the equivalence testing procedures and hence on the outcome of such tests. There are other aspects of the proposed tests of equivalence that give cause for concern but it is impossible to evaluate these properly until the issue of interactions has been resolved.

We therefore fail to see how the proposed tests of difference and equivalence can be regarded as being fit for purpose until the issue of interactions with site has been adequately addressed, and our opinion is that this must be clarified *before *any such methodology becomes a regulatory requirement. We also question the value of trying to evaluate the statistical properties of the proposed methodology prior to this, noting that the authors' simulation studies that purport to establish validity of their methods were performed under a model that implicitly assumes that all potential interactions are non-existent. Whether a valid and workable solution that takes proper account of interactions actually exists within the authors' proposed framework is uncertain.

Whilst the authors make it clear that, in their opinion "The primary objective for an average difference/equivalence approach is neither the identification of possible interactions nor per site (per year) evaluation," this does not in any way lessen the need to partition the sums of squares and degrees of freedom in a way that is consistent with the design structure. The fact that the authors later choose to include site and interaction terms as fixed effects to check for consistency over sites is largely irrelevant here in that it does not address the need to take proper account of interactions in the main difference and equivalence tests. This approach is also questionable given that sites were originally specified as random effects. Furthermore, the way in which interactions with site are handled has the potential to impact on the suitability of different experimental design options, which is of particular concern to companies that are required to implement the proposed methodology. While EFSA's Scientific Opinion document proposes that different reference varieties can be grown at different sites, it is currently unclear which of the possible design options will allow the necessary statistics to be generated when interactions between site and the various genotype subgroups are included in the model.

Irrespective of the specific technical concerns raised above, we see the whole process as extremely convoluted and by no means intuitive. With regard to the authors' proposed equivalence tests, comparisons with traditional equivalence testing are not very helpful in this respect because, with a traditional equivalence test, the focus is on whether the difference between two specific treatments is less than some pre-specified amount whereas here the focus is on the difference between GMO and some hypothetical population of non-GM varieties. This is discussed in more detail by Herman et al (2010) [3].

### Other key concerns

Under the current proposals, the thresholds for the tests of equivalence are entirely study-specific, being totally dependent on the precise set of reference varieties that happened to be included. This being the case, the situation could easily arise in which two different submissions with very similar profiles in terms of GMO, comparator, environments and levels of residual variation could result in very different overall conclusions simply because the respective sets of reference varieties led to very different sets of equivalence limits. Yet this would clearly be inappropriate because both sets of reference varieties are intended to estimate exactly the same thing, i.e. the true spread of responses amongst the entire population of non-GM varieties with a history of safe use grown under identical environments as the test entries. In practice, this problem will be exacerbated by the fact that applicants' access to a diverse range of varieties is often restricted. The need for a level playing field should surely be a key requirement of any regulatory process, and the idea of establishing a common set of equivalence limits should be seriously considered, either based on historical data or, if necessary, based on data from new studies set up specifically for this purpose.

We see no scientific justification for changing the Type I error rate associated with the difference test from the customary 5% level to 10%, as proposed.

## Conclusions

Taking proper account of natural variation amongst commercial varieties in the safety assessment of GM plants makes good sense, but unfortunately the methodology described in van der Voet et al (2011) is seriously flawed and cannot be considered fit for purpose in its current form. Of immediate concern is the need to address the issue of interactions with site. With regard to the difference test, this is not a problem conceptually but implementation remains a challenge currently. With regard to the proposed tests of equivalence, the authors need to firstly explain how they believe interactions should be incorporated conceptually and then demonstrate that this can actually be achieved in practice. Given that the way in which interactions with site are handled will likely impact on the suitability of different experimental design options, absence of this information makes trial planning difficult for those who are expected to implement this methodology.

Even if the issues with interactions are resolved, the suitability of the proposed methodology remains doubtful given that the thresholds for the tests of equivalence would still be entirely study-specific (i.e. similar composition results for a new GM crop could be considered equivalent to the non-GM reference population in one study, and non-equivalent in the next based on the arbitrary sets of reference varieties chosen).

The issues at stake here go far beyond academic interest. They are central to the GM crop regulatory process within the EU because EFSA has made clear that it now expects applicants to adopt this methodology.

## Methods

### Simulation to investigate properties of the difference test when site × genotype interaction is taken into account

**Figure 1:**
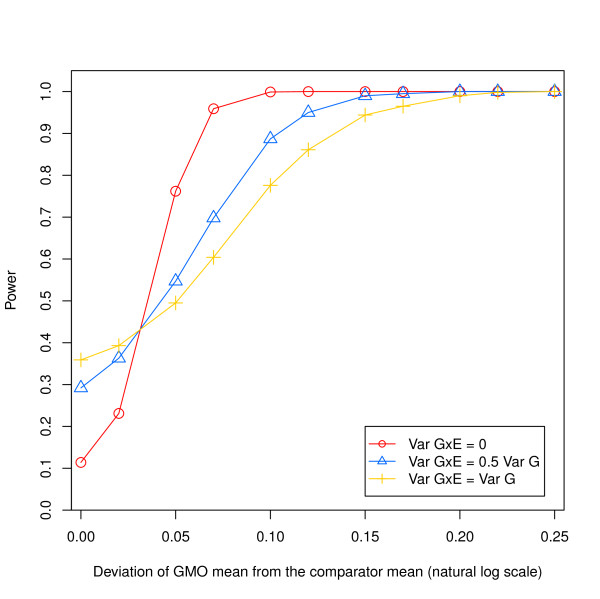
**Estimated power of the difference test assuming different levels of site × genotype interaction**: (a) no interaction, consistent with van der Voet et al. (2011), (b) interaction variance set to be half the size of the genotype variance, (c) interaction variance set to be equal to the genotype variance.

In keeping with van der Voet et al. (2011), data were considered on the log scale. For simplicity, data were generated under the basic model structure in equation (1) with a single component, $\sigma_{SitexGenotype}^{2}$, to represent the entire site × genotype interaction. Values of the other parameters of the model were identical to those in van der Voet et al. (2011), with the exception of *μ_GMO _*where a different spacing was used (i.e. *μ_GMO _*= 0, 0.02, 0.05, 0.07, 0.1, ..., 0.25) to better display the power curves in question. Results were generated for three levels of interaction. The first case, $\sigma_{SitexGenotype}^{2}=0$, is equivalent to the situation assumed by van der Voet et al. (2011) to generate the power curve of the difference test in their Figure Two. Values of $\sigma_{SitexGenotype}^{2}$ in the second and third cases are based on a survey of proprietary compositional data for non-GM varieties from trials on three crops (corn, soybean and canola) with at least 70 analytes per crop, leading us to set the interaction variance to be either half the size of the genotype variance for simulation case 2, or equal to the genotype variance for case 3. The statistical analysis and difference test performed on each of the 1000 data sets generated for each set of parameter values followed the method exactly as proposed by van der Voet et al. (2011). Results are presented in Figure 1.

Empirical estimates of power for the zero-interaction case agree closely with values for the difference test read from Figure Two in van der Voet et al. (2011). In the presence of a site × genotype interaction, however, results are markedly different. Most importantly, in the null situation where *μ_GMO _*= *μ_Control_*, the rejection rate (i.e. false positive error rate) of the difference test is unacceptably high. For tests of significance at the nominal 0.1 level, the estimated rejection rates ± standard error are 0.29 ± 0.014 and 0.36 ± 0.015, when $\sigma_{SitexGenotype}^{2}$ is 0.5x and 1x the magnitude of $\sigma_{Genotype}^{2}$, respectively. In practice, the actual false positive error rate would depend on the relative sizes of the various sub-components of site × genotype interaction.

## Competing interests

All authors are either employees of, or represent, companies that develop and market agricultural products, including transgenic crops.

## Authors' contributions

KJW, CB, BH and MAN were largely responsible for developing the statistical arguments, with input from RAH. BH conducted the simulation, and results were confirmed independently by CB and KJW. All authors contributed to drafts, and read and approved the final manuscript.

## Response

By Hilko van der Voet, Joe N. Perry, Billy Amzal, Claudia Paoletti

Emails: Hilko van der Voet, hilko.vandervoet@wur.nl; Joe N Perry, joe.perry@rothamsted.ac.uk; Billy Amzal, Billy.Amzal@la-ser.com; Claudia Paoletti, Claudia.PAOLETTI@efsa.europa.eu

The comments of Ward et al. on our paper (1) illustrate the importance of taking account of natural variability to an appropriate safety assessment for a GM plant. In our response, we will first focus on areas of agreement, then areas of disagreement, and end with issues requiring clarification or further development. We will not discuss experimental design here, as our paper was restricted to a model for statistical analysis, and explicitly excluded a discussion or definite proposals for experimental design (1, see p.2).

## Areas of agreement

Ward et al. agree with us that "taking proper account of natural variation amongst commercial varieties (genotypes) in the safety assessment of GM plants makes good sense". Ward et al. do not express any explicit disagreement with the general form of statistical modelling, using linear mixed models for appropriately transformed outcomes, where the GM genotype is compared to appropriate comparator and reference varieties. The approach taken by van der Voet et al. (1), that partitions differences between the GM plant, comparator and reference varieties (i.e. the overall genotype variation) into separate components, differs from previous statistical assessment methods in which the variance component between reference varieties was not estimated (e.g. [4-6]). We agree with Ward et al.'s statement that "partitioning the interaction between site and genotype into the corresponding components is the logical next step, supported by the argument that interaction with site may be different among the test entries than among reference varieties".

van der Voet et al. (1) is based on the longer EFSA reportpublished in 2010 (2). EFSA (2) makes clear in two separate sections that analysis "should allow for the possibility of checking for possible site-specific effects, i.e. genotype × site interactions" and that details should be given of the "results of any [such] test of interaction between the test materials and sites". We agree with Ward et al. that it is necessary to take "proper account of interactions in the statistical model", and that there is a "need to address the issue of interactions with site". Whereas our paper did not focus primarily on the issue of interaction, in the Discussion section we referred to individual (site) equivalence as an alternative to average equivalence, and stated that "In the linear mixed model approach the genotype by environment interaction would have to be estimated". Also, while restricting the main analysis in the paper to a model without interaction, we gave a simple example of how "the site by genotype interaction can be investigated" and reported 8 out of 53 analytes to have a significant (p < 0.05) genotype by environment interaction (1, p. 19). The code for this analysis was published as supplementary material (available at http://www.efsa.europa.eu/en/scdocs/doc/1250ax2.pdf) to the longer report (2) on which the paper was based. We also agree that "the way interactions are handled will have a direct bearing on the various standard errors and intervals that are central to the equivalence testing procedures and hence on the outcome of such tests" and that the interaction term should be partitioned into sub-contrasts "in a way that is consistent with the design structure". However, we did not specify how subsequent tests of equivalence should be performed if interactions were found, because this requires careful consideration, in general and on a case-by-case basis (see below).

We also agree that interaction will have a different effect on interpretation depending on which specific contrasts are significant, as Ward et al. stated: "interaction with site may be different among the test entries than among reference varieties." Hence, it is unsurprising that, by analogy with the simulations in our paper, Ward et al. show that when there is actually a single overall interaction variance component present, the false positive rate of the difference test from a model without interaction will be too high and the power of the test diminishes. Such a result is expected because the structure of the simulated data differs from that of the model used for analysis.

## Areas of disagreement

Ward et al. present two key concerns, on which we do not agree. Below we respond to each of these concerns.

### Interactions in the statistical model

Ward et al. state that the issue of interaction with site "must be clarified *before *(their italics) any such methodology [using tests of difference and equivalence] becomes a regulatory requirement". Their critique is that the role of interactions in equivalence testing used for GM plant risk assessment is not yet fully elaborated. First, this critique is misdirected because we acknowledged this same fact already in our paper, e.g. where we wrote (1): "However, the consequences for safety assessment are still unclear, for example would it be possible to declare a GMO equivalent in some environments and not in others?". Therefore, the approach in our paper should be considered as a system within which both simple and more complex analyses may be explored. The provision of a difference test, used traditionally, remains; the addition of an equivalence test provides a robust framework to allow for a proper quantification of natural variation.

Second, adding interactions to the linear mixed model is technically not complex, and we have presented results from a simple model with interaction in our paper. But exactly how to interpret equivalence in the presence of significant interaction requires careful consideration, in general and on a case-by-case basis. It depends for example upon the question whether the investigators are seeking to show equivalence regionally or across all sites, the design of the experiment, the degree to which sites and reference varieties are orthogonal in the design, the form of the statistical mixed model and the variation expressed by the components of the interaction. We therefore do not agree with Ward et al. that proposals for handling difference and equivalence tests, setting equivalence limits based on observed natural variation, and displaying the combined results in a graphical form should be deferred "until the issue of interactions has been resolved".

Ward et al., referring to Herman et al. [3], reject the derivation of our proposed equivalence test claiming that "with a traditional equivalence test, the focus is on whether the difference between two specific treatments is less than some pre-specified amount whereas here the focus is on the difference between GMO and some hypothetical *population *[our italics] of non-GM varieties". Indeed. it was exactly this change of focus that led us to construct a linear mixed model in which the variance within the population of non-GM varieties (V_g_) is included explicitly in the baseline variation when setting equivalence limits (see for example equations 3b and 3d in our paper, for a simple balanced case). In this way, we modified the traditional equivalence test for cases where the reference is a population, rather than a simple treatment. (It may be that Ward et al. have partly been misled in their judgment by an error in our paper which unfortunately occurred outside our control during the final phase of publishing. We corrected this almost immediately by a comment which accompanies the paper on the *BMC Biotechnology *website, but take the opportunity to reproduce it here again, officially: "In the section Methods, subsection Linear mixed models, there is a numbered list summarizing the appropriate calculations for performing the difference and equivalence tests.Under numbers 2 and 4 in this list, the first argument of the *lsd *function in the equations for the confidence limits should be *GR *instead of *GC*." (1, p. 16)).

The choice for treating site as a random or fixed effect was deliberately left open in the Methods section of the paper (1, p 15, "depending on the details of the experimental design") and reflects the flexibility emphasised and described also in EFSA (2). For the specific example we chose to consider site as a random factor. However, we fitted an interaction model involving site as a fixed factor, for the purpose of presenting tables with site-specific means. We did this in GenStat, but it should also be no problem in other statistical packages. This formulation produced the intended division of degrees of freedom, with 3 df for the genotype × environment interaction of two genotypes (GMO and comparator) and 4 sites. In any event, the resulting Wald statistic for the interaction is the same, whether site is taken random or fixed.

### Study-dependent limits of equivalence

Ward et al. consider the use of study-specific equivalence limits as doubtful. An alternative is to have equivalence limits fixed before the study. Equivalence testing using fixed equivalence limits was indeed used by Oberdoerfer et al. [7]. However, Hothorn and Oberdoerfer [8] later described this fixed value method as rigid and not reflecting the difference in variability between analytes, and stated (p. 131) that "In practice, the best approach is the definition of component-specific safety ranges proportional to the component-specific variance of the non-transgenic concurrent control grown in the same field trials". Ward et al. prefer "a common set of equivalence limits, either based on historical data or, if necessary, based on data from new studies set up specifically for this purpose." Firstly, historic data such as that in the ILSI database, to which they refer, give no information concerning natural variation, or the design of the experiments from which values are derived, or the possible correlations between endpoints. Furthermore, no statistical use of the data is possible because there is no information in the database that allows the separation of environmental from genotypic variation. And, whilst we agree with Ward et al. that treatment effects may vary across sites, the logical consequence of this is that only data obtained concurrently from reference varieties randomized in the same experiment as the test genotypes can supply the requisite baseline data for comparison of the GM genotype that accounts for natural variation between sites. The Ward et al. position is therefore logically inconsistent with the agreed need to take "proper account of natural variation amongst commercial varieties and test materials". Secondly, if it is unnecessary to include reference varieties concurrently in the same experiment, as Ward et al. imply, why is it the case that, for at least the last ten years, reference varieties have been included in experiments for compositional trials (see, for example, [9-11] for a review)? Thirdly, the approach cited by Ward et al. of Herman et al. [3] is unacceptable: it fails to identify environmental variation or to separate it from genotypic variation, and would make it easier to prove equivalence between genotypes in small datasets.

We disagree with the claim of Ward et al. concerning different overall conclusions for GM plants with similar profiles. It is a fundamental tenet of statistical theory that different outcomes occur under replication of the experiment, just by chance. However, this alone provides no support to their claim, unless supplemented by additional evidence, which they fail to provide. We think that equivalence limits should be based on the best data available, derived from specific experiments designed to provide the appropriate information. If such studies are done with sufficient scientific rigour, the inevitable statistical fluctuations are not an objection for a proper use of study-specific limits.

In summary, we do not agree with Ward et al. that the methodology described in our paper is either "flawed" or "unfit for purpose".

## Areas requiring clarification or further development

In line with EFSA (2), in our paper the "focus is on easily understood cases" but in which it is implicit that "the statistical approaches presented ... should be adapted in more complex situations". However, in such complex situations there remains the issue concerning how to interpret equivalence tests if interactions are found. The implementation of the proposed approach over time will allow a robust evaluation of the applicability of our methodology to the full range of cases where significant genotype × environment interactions are found. Specifically, this will result in the development of the interpretation of equivalence tests in such cases. Indeed, it might well prove illuminating to present and compare analyses based on models including and excluding genotype × environment interactions. This would in effect encompass a form of sensitivity analysis that would answer some of the issues of robustness raised by Ward et al.

Our paper raised a related issue: namely, "would it be possible to declare a GMO equivalent in some environments and not in others?" This might certainly be a reasonable conclusion to draw from analyses in which significant interactions are demonstrated. One possibility for further analysis might be to perform the difference and equivalence tests separately for subdivisions of the sites such that no large interaction is found within each subdivision.

We miss in the comments of Ward et al. any constructive proposal for how to perform the safety assessment. Criticising an approach is sometimes useful, but much could be learned by trying to integrate the good aspects of different points of view. Further collaboration between statisticians and scientists confronted with these issues would be very helpful to further progress the science behind GMO risk assessment.
